# Co Cluster-Modified Ni Nanoparticles with Superior Light-Driven Thermocatalytic CO_2_ Reduction by CH_4_

**DOI:** 10.3390/molecules29225338

**Published:** 2024-11-13

**Authors:** Mei Li, Yuhua Zhang, Na Sun, Dan Cheng, Peng Sun, Qian Zhang

**Affiliations:** 1 School of Life Science and Technology, Shandong Second Medical University, Weifang 261053, China; limei@sdsmu.edu.cn; 2 School of Pharmacy, Shandong Second Medical University, Weifang 261053, China; zhangyh@sdsmu.edu.cn (Y.Z.); sunna@sdsmu.edu.cn (N.S.); chengdan@sdsmu.edu.cn (D.C.); pengs1993@sdsmu.edu.cn (P.S.)

**Keywords:** light-driven thermocatalysis, CO_2_ reduction, Ni-based catalyst, Co cluster-modified, carbon deposition resistance

## Abstract

Excessive fossil burning causes energy shortages and contributes to the environmental crisis. Light-driven thermocatalytic CO_2_ reduction by methane (CRM) provides an effective strategy to conquer these two global challenges. Ni-based catalysts have been developed as candidates for CRM that are comparable to the noble metal catalysts. However, they are prone to deactivation due to the thermodynamically inevitable coking side reactions. Herein, we reported a novel Co-Ni/SiO_2_ nanocomposite of Co cluster-modified Ni nanoparticles, which greatly enhance the catalytic durability for light-driven thermocatalytic CRM. It exhibits high production rates of H_2_ (*r*_H2_) and CO (*r*_CO_, 22.8 and 26.7 mmol min^−1^ g^−1^, respectively), and very high light-to-fuel efficiency (*ƞ*) is achieved (26.8%). Co-Ni/SiO_2_ shows better catalytic durability than the referenced catalyst of Ni/SiO_2_. Based on the experimental results of TG-MS, TEM, and HRTEM, we revealed the origin of the significantly enhanced light-driven thermocatalytic activity and durability as well as the novel photoactivation. It was discovered that the focused irradiation markedly reduces the apparent activation energy of CO_2_ on the Co-Ni/SiO_2_ nanocomposite, thus significantly enhancing the light-driven thermocatalytic activity.

## 1. Introduction

The rapid consumption of fossil fuels accompanied by population growth not only exacerbated the energy crisis, but also caused the excessive emissions of CO_2_ (greenhouse gas), leading to a critical greenhouse effect [1,2]. The photocatalytic reduction of CO_2_ driven by inexhaustible solar energy was an effective solution to the above-mentioned issues, which attracted wide attention [3,4,5,6,7]. However, the photocatalytic approach suffers from a low fuel production rate and low light-to-fuel efficiency (*η*).

In recent years, light-driven thermocatalytic (photothermocatalytic) CO_2_ reduction with CH_4_ [8,9,10,11,12,13,14,15,16,17,18,19], H_2_ [20,21] and H_2_O [22,23,24] has been considered as a promising approach, as it takes into account the advantages of the low energy consumption of photocatalysis and the high catalytic activity of thermocatalysis. Among several CO_2_ reduction approaches, light-driven thermocatalytic CRM is a prospective option on account of the high conversion rate of CO_2_ and CH_4_ to syngas and the achievement of solar-to-chemical energy conversion [25,26,27,28,29,30,31,32,33,34,35]. In the meantime, VIII group metal catalysts were reported to exhibit catalytic activity for light-driven thermocatalytic CRM. Among the catalysts, non-precious metal catalysts, such as Ni-based catalysts, have attracted great interest as a result of their low price, availability, and superior initial activity in contrast to the noble metal catalysts [8,9,18,19,36,37,38]. However, Ni-based catalysts are prone to severe deactivation which restrict their practical applications. As the coking deposition side reactions (CO disproportionation and CH_4_ dissociation) are thermodynamically favorable [39,40,41], the immediate concern is to exploit new Ni-based catalysts that can dynamically suppress carbon deposition. Therefore, these great challenges for the rational design of Ni-based catalysts are urgent to be tackled. Recently, there are several approaches that have been provided to significantly inhibit the carbon deposition on Ni-based catalysts [11,15,42,43,44,45,46,47], including modifying the surface of Ni nanoparticles with MgO clusters [42] or CeO_2_ clusters [43], by which the oxidation of carbon species can be accelerated.

Herein, we prepared a Co-Ni/SiO_2_ nanocomposite of Co cluster-modified Ni nanoparticles, which greatly boost the activity and durability of the nanocomposite for light-driven thermocatalytic CRM. It exhibits high production rates of H_2_ (*r*_H2_) and CO (*r*_CO_, 22.8 and 26.7 mmol min^−1^ g^−1^, respectively). The light-to-fuel efficiency (*η*) of the catalyst is as high as 26.8%. The high yield production rate originates from effective photothermal conversion and photoactivation induced by light irradiation. It was found that Co cluster modification of the Ni nanoparticles considerably enhances their light-driven thermocatalytic durability. Based on the experimental results, we delved into the origin of the excellent durability and photoactivation in the nanocomposite.

## 2. Results and Discussion

### 2.1. Catalyst Characterization

**Figure 1:**
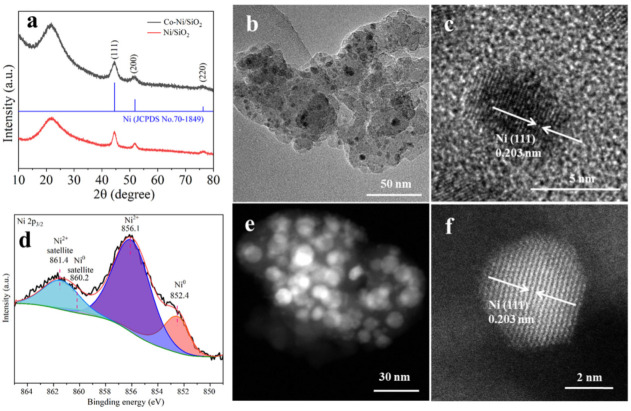
(**a**) XRD pattern, (**b**,**c**) TEM and HRTEM image, (**d**) XPS spectra of Ni 2p_3/2_, and (**e**,**f**) HAADF-STEM image of Co-Ni/SiO_2_.

ICP (Inductively Coupled Plasma) analysis revealed that the Ni loadings of Co-Ni/SiO_2_ and Ni/SiO_2_ were 10.73 and 9.89 wt%, respectively. The XRD (X-ray diffraction) pattern showed that Ni exists in a crystalline metallic Ni phase (PDF70-1849) both in Co-Ni/SiO_2_ and Ni/SiO_2_, while silica exists in an amorphous phase (Figure 1a). No diffraction peaks for Co species can be observed in Co-Ni/SiO_2_, which is mainly ascribed to the low content or the amorphous status of the Co species. The average diameters of the metallic Ni nanoparticles in Co-Ni/SiO_2_ and Ni/SiO_2_, calculated according to the Scherrer formula (L = 0.89λ/βcosθ), were 4.5 and 4.1 nm, respectively. The TEM (Transmission Electron Microscopy) image (Figure 1b) and HAADF-STEM image (Figure 1e) showed that Ni nanoparticles were well dispersed on the amorphous silica in Co-Ni/SiO_2_. HRTEM (High-Resolution Transmission Electron Microscopy) (Figure 1c) and high-resolution HAADF-STEM (High-Angle Annular Dark-Field Scanning Transmission Electron Microscopy) (Figure 1f) images show that the Ni nanoparticles with a lattice spacing of 0.203 nm corresponding to the (111) facets were surrounded by the amorphous silica, but no lattice fringe attributed to the Co nanoparticles can be found in the field. HAADF-STEM images with element mapping were used to characterize the element distribution of Ni, Co, Si, and O in the Co-Ni/SiO_2_ sample. As shown in Figure S1, the Ni nanoparticles were surrounded by Co clusters and uniformly distributed on the SiO_2_. This result, together with the XRD results, indicate that Co species exist in an amorphous state. The TEM and HRTEM images of Ni/SiO_2_ showed that the Ni nanoparticles were well dispersed on the SiO_2_ with a lattice spacing of 0.203 nm corresponding to the (111) facets (Figure S2). XPS (X-ray photoelectron spectroscopy) showed that Ni presents as Ni^2+^ and Ni^0^ species in Co-Ni/SiO_2_ (Figure 1d). The presence of Ni^2+^ was ascribed to the oxidation of Ni species by the air, and the molar ratio of Ni^0^/Ni^2+^ was estimated to be 0.26. Si and Co exist in the form of Si^4+^ and Co^0^ (Figure S3), and the slight positive shifts of the Co^0^ peaks in binding energy were mainly attributed to the strong electron interactions of the closely contacted Ni and Co (Figure S3b). The N_2_ adsorption studies showed that the Brunauer–Emmett–Teller (BET) surface area of Co-Ni/SiO_2_ and Ni/SiO_2_ were 286.8 and 297.2 m^2^ g^−1^, respectively (Figure S4).

### 2.2. Light-Driven Thermocatalytic CRM Activity

**Figure 2:**
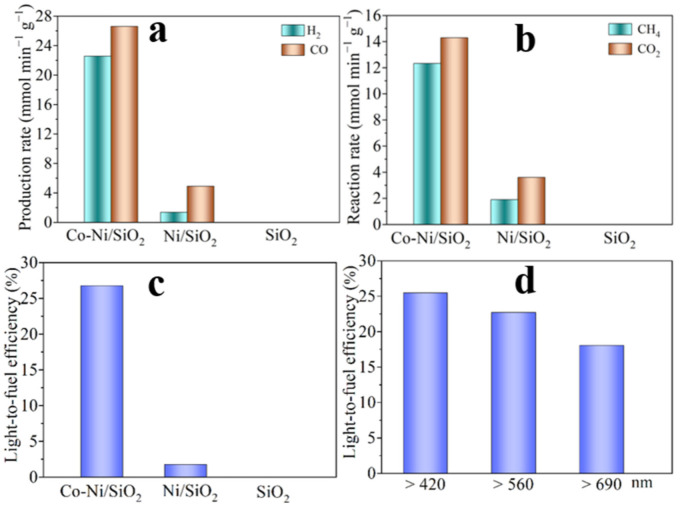
(**a**) The specific reaction rate of CH_4_ and CO_2_, (**b**) the specific production rate of H_2_ and CO, (**c**) the light-to-fuel efficiency for light-driven thermocatalytic CRM under focused UV-Vis-IR irradiation, and (**d**) the light-to-fuel efficiency for the light-driven thermocatalytic CRM of Co-Ni/SiO_2_ under the vis-IR light or infrared light from the Xe lamp.

The light-driven thermocatalytic CRM activity of the catalysts were conducted in a hand-made reactor with a quartz window as shown in Figure S1. The heat source was provided by a 500 W Xe lamp. Pure SiO_2_ has no catalytic activity for CRM, while Co-Ni/SiO_2_ exhibits extraordinary catalytic activity under focused UV-Vis-IR irradiation (Figure 2a). The H_2_ and CO production rates (*r*_H2_, *r*_CO_) of the Co-Ni/SiO_2_ catalyst reached 22.8 and 26.7 mmol min^−1^ g^−1^, respectively (Figure 2b), which are higher than that of Ni-/SiO_2_ (*r*_H2_ and *r*_CO_ were 1.4 and 4.9 mmol min^−1^ g^−1^, respectively).

CRM is a strongly endothermic reaction (Δ*H*_298_ = 247.0 KJ mol^−1^). The high *r*_H2_ and *r*_CO_ values of Co-Ni/SiO_2_ indicate that efficient light-to-fuel conversion occurred as the light-driven thermocatalytic CRM is merely driven by focused UV-Vis-IR irradiation.

The light-to-fuel efficiency (*η*) of Co-Ni/SiO_2_ for CRM under focused UV-Vis-IR irradiation reached a high value of 26.8% (Figure 2c), which is far higher than that of Ni/SiO_2_ (1.8%). In addition, Co-Ni/SiO_2_ also showed high catalytic activity for CRM under visible-infrared or infrared irradiation (Figure S5). Under vis-IR irradiation (λ > 420 nm), Co-Ni/SiO_2_ showed higher *r*_H2_ and *r*_CO_ values of 16.4 and 21.2 mmol min^−1^ g^−1^, with an *ƞ* of 25.5%. Even under focused infrared irradiation (λ > 690 nm), Co-Ni/SiO_2_ still demonstrated good catalytic CRM activity, with an *η* of 18.1% (Figure 2d).

### 2.3. Light-Driven Thermocatalytic Durability

**Figure 3:**
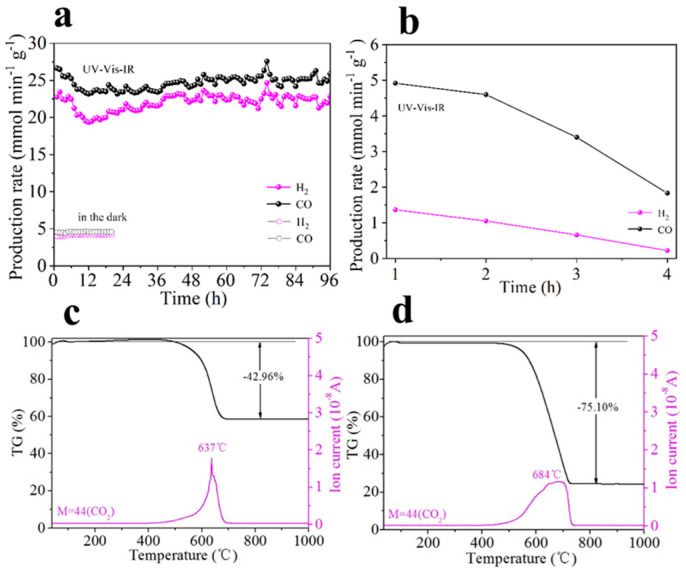
(**a**) The time course of the reaction and production rates for Co-Ni/SiO_2_ with focused UV-Vis-IR irradiation and in the dark. (**b**) The time course of the reaction and production rates for Ni/SiO_2_ with focused UV-Vis-IR irradiation, TG-MS profiles for the used Co-Ni/SiO_2_ (**c**) and Ni/SiO_2_ (**d**) samples after the durability tests.

The long-term light-driven thermocatalytic CRM durability of Co-Ni/SiO_2_ was performed under focused UV-Vis-IR irradiation. As shown in Figure 3a, the *r*_H2_ and *r*_CO_ at 96 h were 22.8 and 25.9 mmol min^−1^ g^−1^, which were identical to the initial activity at 1 h (Figure 3a). This result indicates that Co-Ni/SiO_2_ exhibits superior light-driven thermocatalytic CRM durability. On the contrary, Ni/SiO_2_ showed much lower catalytic activity than Co-Ni/SiO_2_ under the same reaction conditions (Figure 3b). Its *r*_H2_ and *r*_CO_ at 1 h were 1.4 and 4.9 mmol min^−1^ g^−1^, respectively. Upon extending the irradiation time to 4 h, the *r*_H2_ and *r*_CO_ quickly decreased to 0.2 and 1.8 mmol min^−1^ g^−1^, respectively. The reason for this rapid deactivation of Ni/SiO_2_ is the carbon (produced by CH_4_ dissociation and CO disproportionation at high temperatures) deposited on the surface of the Ni nanoparticles, which covered the active sites.

### 2.4. Origin of the Superior Light-Driven Thermocatalytic Durability

To delve into why the Co-Ni/SiO_2_ nanocomposite exhibits superior light-driven thermocatalytic CRM durability, TG-MS (Thermogravimetric Mass Spectrometry), TEM, and HRTEM were employed to characterize the used Co-Ni/SiO_2_ and Ni/SiO_2_ samples after the durability tests. As shown in the TG-MS profiles, the weight loss of the Co-Ni/SiO_2_ and Ni/SiO_2_ samples were 42.96 and 75.10%, respectively (Figure 3c,d), owing to the combustion of carbon. The rate of carbon deposition (*r*_C_) for the Co-Ni/SiO_2_ and Ni/SiO_2_ samples were calculated to 1.25 × 10^−2^ and 0.23 g_C_ g_catal_^−1^ h^−1^, respectively. The results of TEM also confirmed that there are more carbon nanofibers that can be observed in the used Ni/SiO_2_ sample than the used Co-Ni/SiO_2_ sample (Figure S6a,c). The HRTEM image showed that there was no carbon deposition on the Ni nanoparticles in Co-Ni/SiO_2_ (Figure S6b). On the contrary, severe deposition of graphite carbon with a lattice spacing of 0.340 nm corresponding to the (002) facets on the surface of Ni nanoparticles in Ni/SiO_2_ can be observed (Figure S6d), leading to the fast deactivation. This reveals the Co cluster modification of Ni nanoparticles in Co-Ni/SiO_2_ could inhibit the carbon deposition on the Ni nanoparticles as compared to Ni/SiO_2_, thus significantly enhancing the light-driven thermocatalytic durability. This result can be attributed to the modification of Ni by the Co cluster, which dilutes the surface atoms of Ni and prevents coke nucleation, making it more difficult for carbon atoms to bond.

### 2.5. The Function of Light

#### 2.5.1. Heating Role

**Figure 4:**
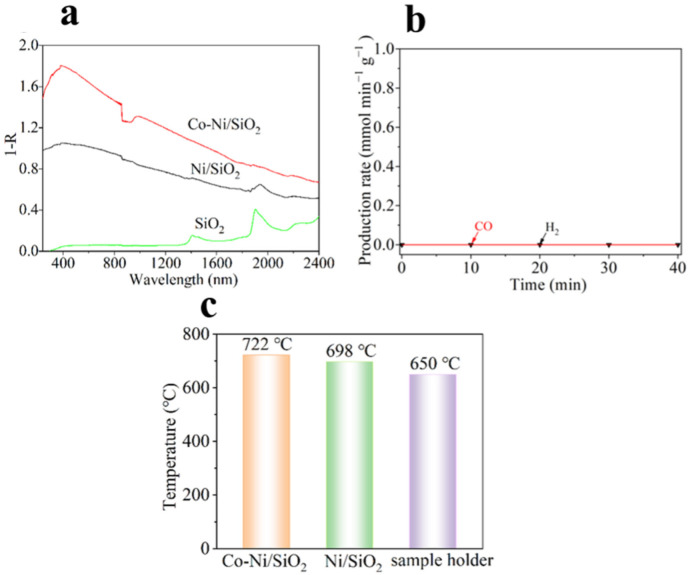
(**a**) The diffuse reflectance UV-vis-infrared absorption spectra of the samples. (**b**) The time course of H_2_ and CO production rates of the Co-Ni/SiO_2_ sample for CRM under focused UV-Vis-IR irradiation at near-room temperature. (**c**) The stable temperature (*T*_st_) of Co-Ni/SiO_2_, Ni/SiO_2_, and the sample holder under focused UV-Vis-IR irradiation.

To reveal the function of focused irradiation during the light-driven thermocatalytic CRM, the optical absorption of Co-Ni/SiO_2_ and Ni/SiO_2_ were measured. As shown in Figure 4a, both Co-Ni/SiO_2_ and Ni/SiO_2_ demonstrate strong absorption from 240 to 2400 nm, which arises from the surface plasma absorption of the metallic Ni nanoparticles [48]. The photocatalytic CRM activity of the Co-Ni/SiO_2_ sample at near ambient temperatures under irradiation was performed. No detectable CO and H_2_ are produced, indicating that the CRM process cannot be driven by light irradiation at near-room temperature (Figure 4b). This result suggests that the highly effective catalytic activity of Co-Ni/SiO_2_ under focused irradiation arises from effective photothermal conversion.

Under focused irradiation, the temperature of the catalysts quickly increased to a steady temperature (*T*_st_) as a result of the surface plasma absorption of metallic Ni nanoparticles and the infrared heating effect. The *T*_st_ values of the Co-Ni/SiO_2_ and Ni/SiO_2_ samples and the empty sample holder were 722, 698, and 650 °C, respectively (Figure 4c). The thermocatalytic CRM then proceeds at a high temperature. The high *T*_st_ value of the empty sample holder suggests that the infrared heating effect has as a critical role in raising the surface temperature of the samples.

Under focused visible-infrared and infrared irradiation (λ > 420, 560, and 690 nm), the Co-Ni/SiO_2_ sample reached its *T*_st_ of 680, 642, and 598 °C, respectively (Figure S7). In addition, the light-driven thermocatalytic CRM can proceed under focused irradiation of UV-Vis-IR, visible-infrared light, and near infrared light.

#### 2.5.2. Photoactivation

To further reveal whether the light plays another role in the light-driven thermocatalytic CRM, the thermocatalytic activity of Co-Ni/SiO_2_ in the dark for CRM was measured at 722 °C as well as the *T*_st_ under focused irradiation. As shown in Figure 3a, its thermocatalytic activity was lower than that of focused irradiation. Under focused light irradiation, the r_H2_ and *r*_CO_ at the first 1 h were promoted by 5.7 and 5.9 times, respectively, in comparison with those in the dark. This result indicates that the light not only plays a heating role but also induces photoactivation as the focused irradiation light can significantly promote the catalytic activity.

**Figure 5:**
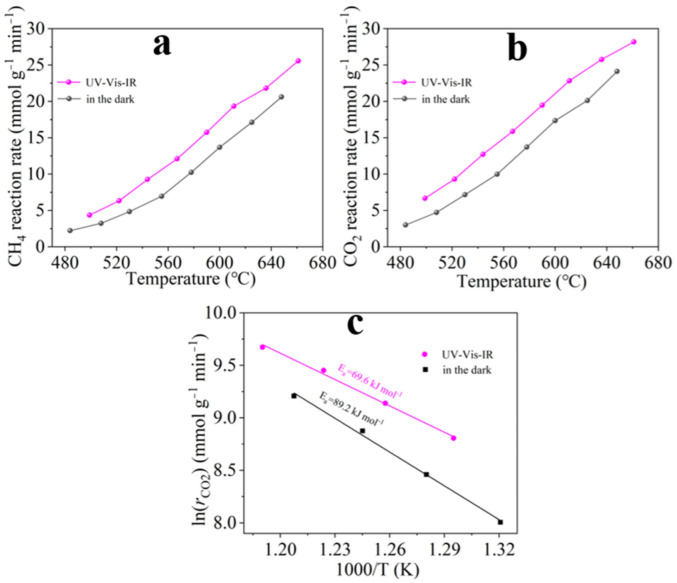
The *r*_CH4_ and *r*_CO2_ of the Co-Ni/SiO_2_ sample for CRM at different temperatures under focused UV-Vis-IR and in the dark (**a**,**b**), and ln(*r*_CO2_) vs. 1/*T* (*C*) for CRM on Co-Ni/SiO_2_ under focused irradiation and in the dark (**c**).

To probe into the photoactivation, the catalytic CRM activity of Co-Ni/SiO_2_ at different temperatures in the dark and under focused UV-Vis-IR irradiation were evaluated. The catalytic CRM activity of Co-Ni/SiO_2_ was greatly enhanced under focused irradiation compared with those in the dark (Figure 5a,b). However, Co-Ni/SiO_2_ has no photocatalytic activity under irradiation at near-room temperature, Therefore, the significant enhancement of the catalytic CRM activity under focused irradiation can be attributed to the photoactivation effect in addition to the efficient photothermal conversion.

To deeply reveal the photoactivation, we drew a diagram of ln(*r*_CO2_) *vs.* 1/*T* based on the *r*_CO2_ data of Co-Ni/SiO_2_ at different temperatures under focused irradiation and in the dark. As shown in Figure 5c, there is a good linear relationship between ln(*r*_CO2_) *vs.* 1/*T*. The apparent activation energy (*Ea*) was calculated by means of the Arrhenius equation (*k* = Ae^−*Ea*/RT^). The *Ea* of CO_2_ on Co-Ni/SiO_2_ was 89.2 kJ mol^−1^ in the dark, but under focused irradiation, the *Ea* of CO_2_ was remarkably reduced to 69.6 kJ mol^−1^. This result demonstrates that photoactivation facilitates the catalytic CRM activity of Co-Ni/SiO_2_ by reducing the *Ea* directly.

## 3. Materials and Methods

### 3.1. Catalyst Synthesis

Co-Ni/SiO_2_ nanocomposite: Na_2_SiO_3_.9H_2_O (25.578 g) and deionized water (85 mL) were stirred for 15 min, then the diluted HNO_3_ solution (the volume ratio of HNO_3_ to deionized water was 1:6) was added dropwise to the mixed solution under magnetic stirring until the vitreous products reached a pH of ~6. A mixed solution of Ni(NO_3_)_2_ (2.6173 g), Co(NO_3_)_2_ (0.2911 g), and deionized water (14 mL) was added dropwise to the vitreous products under magnetic stirring, then the aqueous ammonia solution (6 mL; the volume ratio of concentrated ammonia solution to deionized water was 1:5) was added dropwise to mixed solution. The mixture was kept at 90 °C for 24 h sealed by polyethylene film after being filtered, washed, dried, and grinded. The obtained powder was pre-reduced with 5.0 vol% H_2_/Ar (25 mL min^−1^) at 700 °C for 1 h to obtain Co-Ni/SiO_2_.

SiO_2_ sample: The pure SiO_2_ sample was prepared using a similar procedure with that of the above Co-Ni/SiO_2_ nanocomposite except that there was no addition of Ni(NO_3_)_2_·6H_2_O and Co(NO_3_)_2_.6H_2_O.

Ni/SiO_2_ sample: The production method of Ni/SiO_2_ sample was consistent with those of Co-Ni/SiO_2_ nanocomposite, except for the aqueous ammonia solution by NaOH aqueous solution (dissolved 0.48 g of NaOH with 10.0 g of deionized water).

### 3.2. Characterization

XRD patterns of the samples were obtained on a Rigaku (Shimadzu, Tokyo, Japan) Dmax X-ray diffractometer equipped with Cu Kα radiation. TEM images and energy-dispersive X-ray spectrographs (EDX) were acquired on a JEM-ARM200F electron microscope (Thermo Fisher Scientifitic, Waltham, MA, USA), while the specific surface areas of the samples were measured by N_2_ adsorption at −196 °C on an ASAP2020 instrument (Micrometer, Saint Louis, MO, USA). XPS spectra were recorded on an ESCALAB 250Xi X-ray photoelectron spectrometer using Mg Kα radiation (Perkin Elmer, Waltham, MA, USA). Thermogravimetric/mass spectrometry (TG-MS) analysis of the used sample after reaction was taken on a NETZSCH (Selb, Germany) STA449F3 thermal analyzer connected to a QMS403 mass spectrometer. Diffuse reflectance UV-visible-infrared absorption (DRUV-Vis-IR) spectra were acquired on a Lambda 750S spectrophotometer (Perkin Elmer, Waltham, MA, USA).

### 3.3. Light-Driven Thermocatalytic and Photocatalytic CRM Tests

The light-driven thermocatalytic CRM activity and durability of the samples for CRM under UV-Vis-IR irradiation from 500 W Xe lamp were assessed in a hand-made reactor equipped with quartz window (Scheme S1). A total of 0.0025 g of the sample was filled into the reactor, and a feed stream of 20.3–20.3–59.4 vol% CH_4_-CO_2_-Ar was flowed into the reactor at a flow rate of 90.5 mL min^−1^ controlled by a gas mass flow meter (S49-31MT). Gas-chromatography (GC-9560) was employed to determine the reactants and products.

The light-to-fuel efficiency (*η*) is calculated as follows [49]:

*η* = (*r*_H2_ × Δ*_c_H*_H2_ + *r*_CO_ × Δ*_c_H*_CO_ − *r*_CH4_ × Δ*_c_H*_CH4_)/*P*_solar irradiation_

The values of Δ*_c_H*_H2,_ Δ*_c_H*_CO_, and Δ*_c_H*_CH4_ were calculated under working temperatures. *P*_solar irradiation_ is the power of the UV-Vis-IR irradiation focused into the reactor. An optical power meter (Newport 1918-R) was used to measure the power of the UV-Vis-IR irradiation into the reactor.

### 3.4. Catalytic CRM Activity in the Dark and Under UV-Vis-IR Irradiation

The catalytic CRM of the sample at different temperatures in the dark or under UV-Vis-IR irradiation was assessed on the reactor equipped with quartz window. The catalyst (0.005 g) was placed into the reactor with a continuous feed stream of 10.5–10.5–79.0 vol% CH_4_-CO_2_-Ar at a rate of 40.3 mL min^−1^, and the electric furnace with temperature control program was used to control reaction temperature in the dark and under UV-Vis-IR irradiation.

## 4. Conclusions

In summary, we designed novel Co cluster-modified Ni nanoparticles on Co-Ni/SiO_2_ nanocomposite for light-driven thermocatalytic CRM under UV-Vis-IR irradiation. The Co-Ni/SiO_2_ nanocomposite displays high H_2_ and CO production (22.8 and 26.7 mmol min^−1^ g^−1^, respectively) and superior catalytic durability under light irradiation. Efficient photothermal conversion originates from the plasma absorption of the Ni nanoparticles, while the infrared heating effect is the origin of the high yield production. Co-Ni/SiO_2_ demonstrates better catalytic durability than the Ni/SiO_2_ catalyst that is attributed to the cluster-modification of Co, which can inhibit coke deposition on the Ni nanoparticles (active sites) in Co-Ni/SiO_2_. The light in the CRM reaction not only acts as a heating source but also induces photoactivation, thus further enhancing the activity by reducing the apparent activation energy of CRM. This study provides helpful insights for rationally designing Ni-based catalysts with good catalytic activity and superior catalytic durability for solar light-driven CO_2_ reduction, thus reducing the greenhouse effect and energy shortage.

## Data Availability

Data are contained within the article and Supplementary Materials; further inquiries can be directed to the corresponding author.

## Supplementary Materials

The following supporting information can be downloaded at https://www.mdpi.com/article/10.3390/molecules29225338/s1, Scheme S1: Structural diagram of home-made stainless steel reactor; Figure S1: Co, Ni, Si, and O HAADF-STEM mapping of Co-Ni/SiO_2_; Figure S2: (a) TEM and (b) HRTEM image of Ni/SiO_2_; Figure S3: (a) Si 2p and (b) Co 2p XPS spectra of Co-Ni/SiO_2_; Figure S4: N_2_ adsorption/desorption isotherms of (a) Co-Ni/SiO_2_ and (b) Ni/SiO_2_; Figure S5: Specific production rate of H_2_ and CO on Co-Ni/SiO_2_ for light-driven thermocatalytic CRM under focused irradiation with wavelengths above 420, 560, and 690 nm; Figure S6: TEM (a) and HRTEM (b) images for used Co-Ni/SiO_2_, and TEM (c) and HRTEM (d) images for used Ni/SiO_2_ samples after light-driven thermocatalytic durability tests; Figure S7: Stable temperature (*T*_st_) of the Co-Ni/SiO_2_ under visible-infrared and infrared irradiation with wavelengths above 420, 560, and 690 nm.
