# Supplementary material for: Co Cluster-Modified Ni Nanoparticles with Superior Light-Driven Thermocatalytic CO2 Reduction by CH4
Source: Molecules. 2024 Nov 13;29(22):5338. doi: 10.3390/molecules29225338 (PMC11596596; doi:10.3390/molecules29225338)
Supplement: Supplementary file 1 [file molecules-29-05338-s001.zip › molecules-3292039-supplementary.pdf]

Supporting Information

Co cluster-modified Ni Nanoparticles with superior light-driven catalytic CO_2 reduction by CH_4

Mei Li [1], Yuhua Zhang [2], Na Sun [2], Dan Cheng [2], Peng Sun 2, Qian Zhang [2,*],

[1] School of Life Science and Technology, Shandong Second Medical University, Weifang, 261053, China

[2] School of Pharmacy, Shandong Second Medical University, Weifang, 261053, China

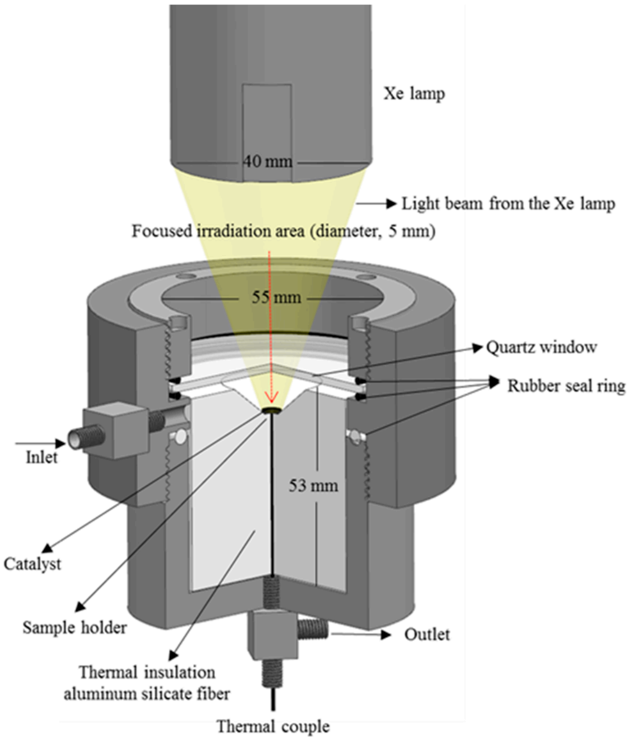

Scheme S1. The structural diagram of home-made stainless steel reactor.

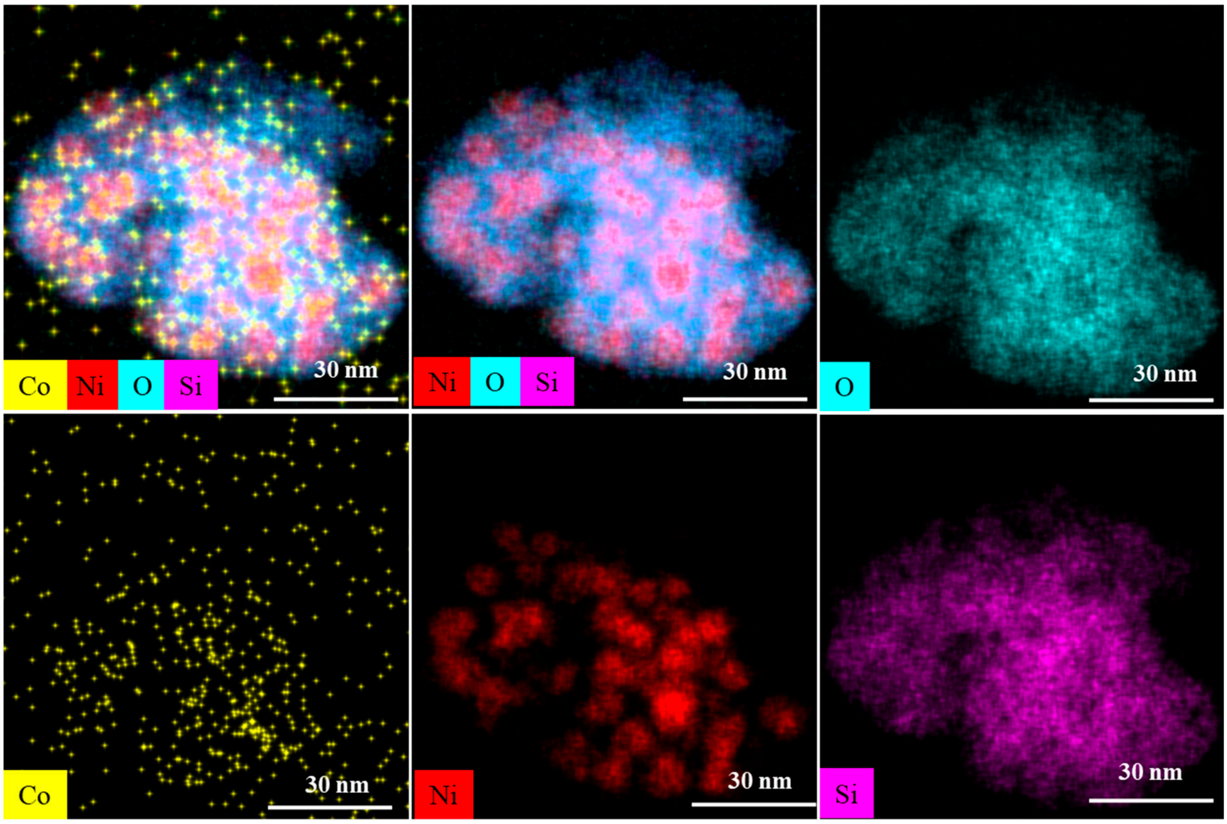

Figure S1. Co, Ni, Si and O HAADF-STEM mapping of Co-Ni/SiO_2.

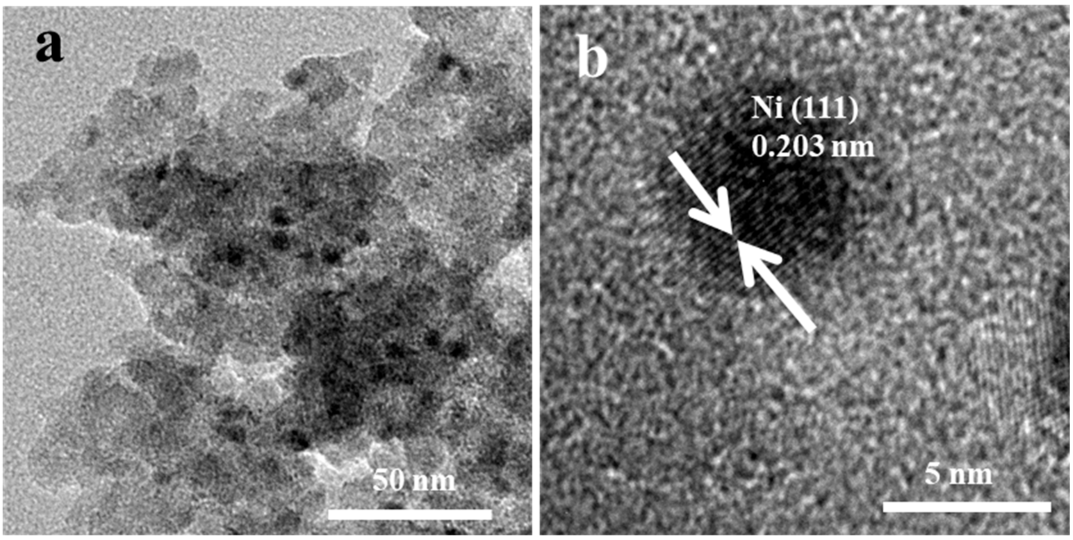

Figure S2. a) TEM and b) HRTEM image of Ni/SiO_2.

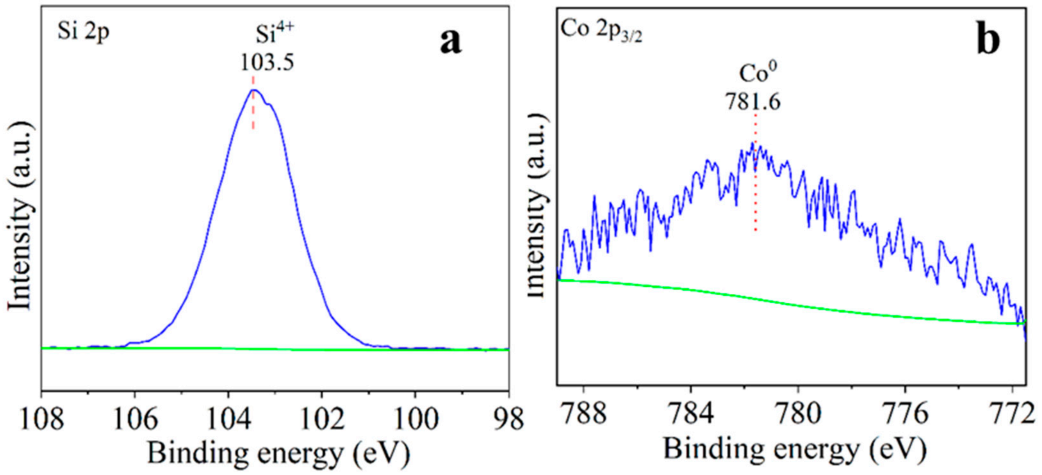

Figure S3. a) Si 2p and b) Co 2p XPS spectra of Co-Ni/SiO_2.

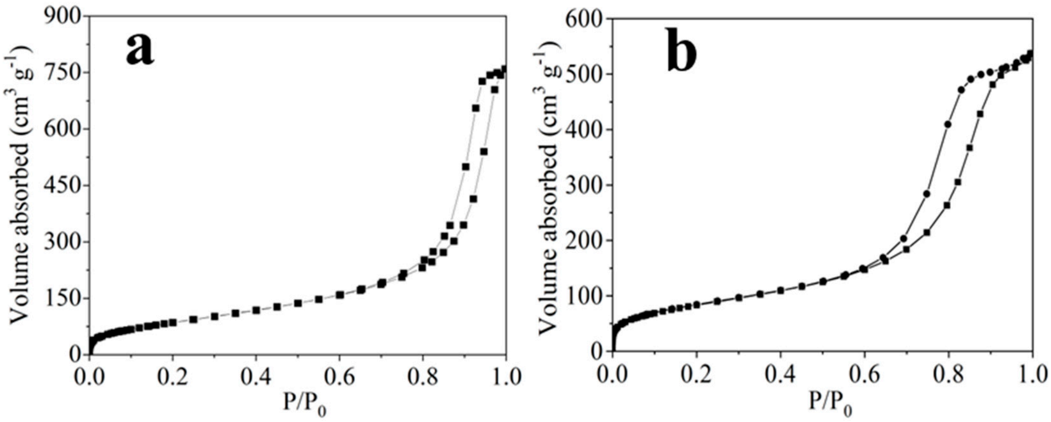

Figure S4. N_2 adsorption/desorption isotherms of a) Co-Ni/SiO_2 and b) Ni/SiO_2.

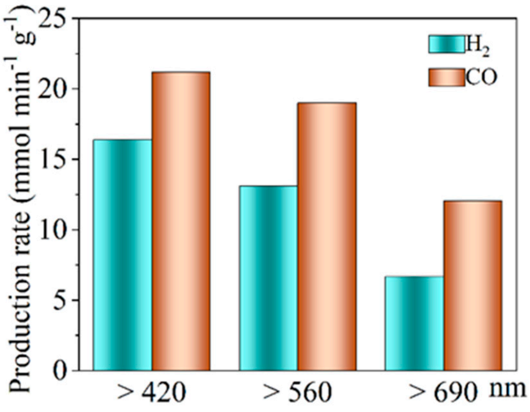

Figure S5. The specific production rate of H_2 and CO on Co-Ni/SiO_2 for light-driven thermocatalytic CRM under the focused irradiation with wavelengths above 420, 560, and 690 nm

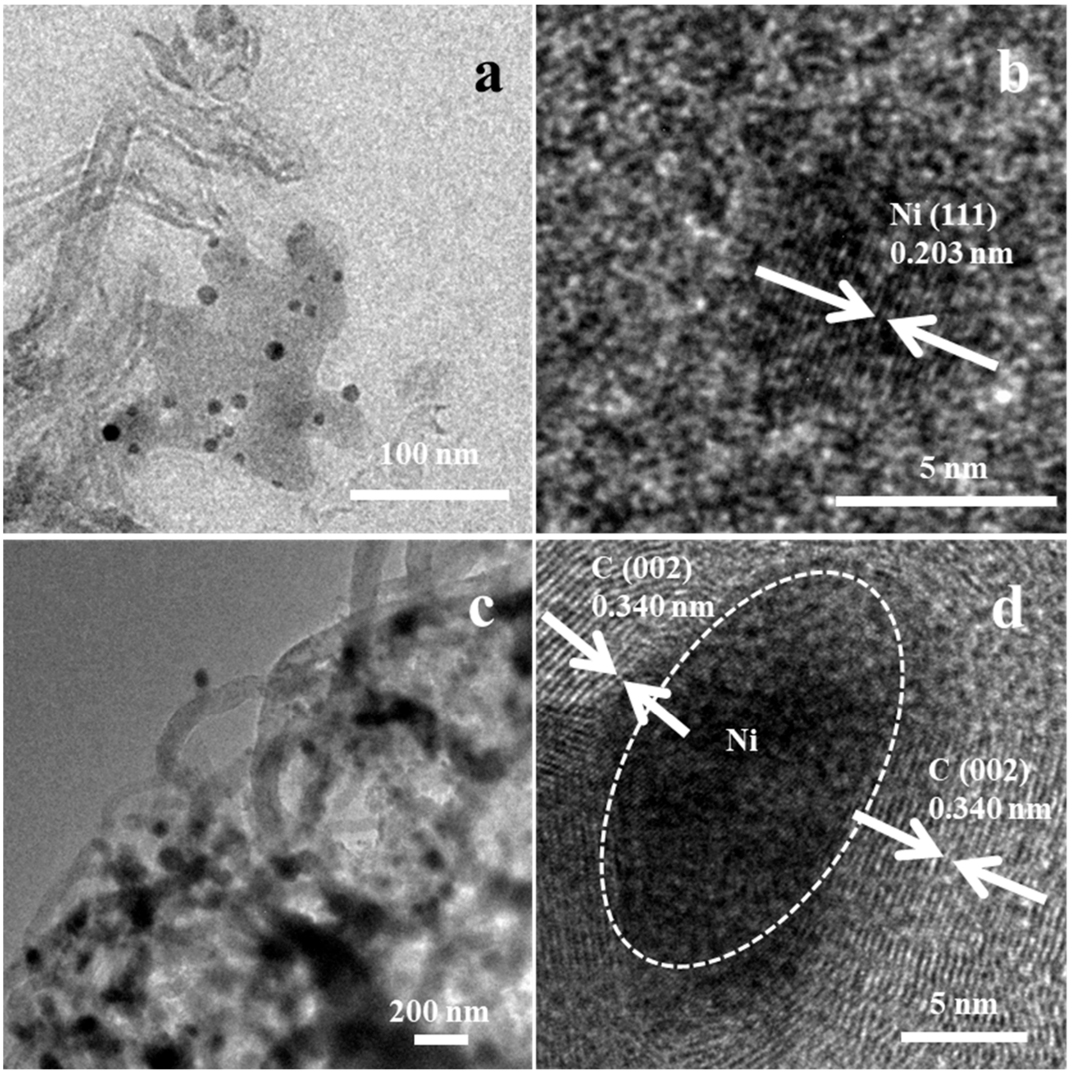

Figure S6. TEM a) and HRTEM b) image for used Co-Ni/SiO_2 and TEM c) and HRTEM d) image for used Ni/SiO_2 samples after the light-driven thermocatalytic durability tests.

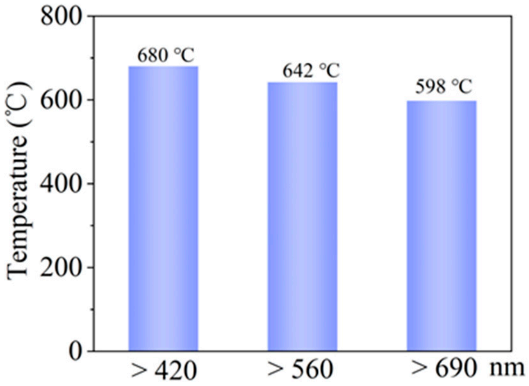

Figure S7. The stable temperature (T_{st}) of the Co-Ni/SiO_2 under the visible-infrared and infrared irradiation with wavelengths above 420, 560, and 690 nm.
